# Neural crest-specific deletion of *Ldb1* leads to cleft secondary palate with impaired palatal shelf elevation

**DOI:** 10.1186/1471-213X-14-3

**Published:** 2014-01-17

**Authors:** Asma Almaidhan, Jeffry Cesario, Andre Landin Malt, Yangu Zhao, Neeti Sharma, Veronica Choi, Juhee Jeong

**Affiliations:** 1 Department of Basic Science and Craniofacial Biology, New York University College of Dentistry, 10010 New York, NY, USA; 2 Consortium for Translational Orthodontic Research, New York University College of Dentistry, 10010 New York, NY, USA; 3 Program on Genomics of Differentiation, Eunice Kennedy Shriver National Institute of Child Health and Human Development, National Institutes of Health, 20892 Bethesda, MD, USA

**Keywords:** Cleft palate, Craniofacial development, LDB1

## Abstract

**Background:**

LIM domain binding protein 1 (LDB1) is a transcriptional co-factor, which interacts with multiple transcription factors and other proteins containing LIM domains. Complete inactivation of *Ldb1* in mice resulted in early embryonic lethality with severe patterning defects during gastrulation. Tissue-specific deletions using a conditional knockout allele revealed additional roles of *Ldb1* in the development of the central nervous system, hematopoietic system, and limbs. The goal of the current study was to determine the importance of *Ldb1* function during craniofacial development in mouse embryos.

**Results:**

We generated tissue-specific *Ldb1* mutants using Wnt1-Cre, which causes deletion of a floxed allele in the neural crest; neural crest-derived cells contribute to most of the mesenchyme of the developing face. All examined *Wnt1-Cre;Ldb1*^*fl/-*^ mutants suffered from cleft secondary palate. Therefore, we performed a series of experiments to investigate how *Ldb1* regulated palate development. First, we examined the expression of *Ldb1* during normal development, and found that *Ldb1* was expressed broadly in the palatal mesenchyme during early stages of palate development. Second, we compared the morphology of the developing palate in control and *Ldb1* mutant embryos using sections. We found that the mutant palatal shelves had abnormally blunt appearance, and failed to elevate above the tongue at the posterior domain. An in vitro head culture experiment indicated that the elevation defect was not due to interference by the tongue. Finally, in the *Ldb1* mutant palatal shelves, cell proliferation was abnormal in the anterior, and the expression of *Wnt5a*, *Pax9* and *Osr2*, which regulate palatal shelf elevation, was also altered.

**Conclusions:**

The function of *Ldb1* in the neural crest-derived palatal mesenchyme is essential for normal morphogenesis of the secondary palate.

## Background

Craniofacial development begins when the cranial neural crest cells (NCCs), which are migratory multipotent precursors, delaminate from the dorsal brain and migrate ventro-laterally to form the mesenchyme of facial primordia, known as the frontonasal prominence and pharyngeal arches [1,2]. The frontonasal prominence develops into the mid- and upper face, while the first pharyngeal arch turns into the lateral skull, most of the jaw, and part of the middle ear. The first pharyngeal arch is further divided into maxillary arch, which is the prospective upper jaw, and mandibular arch, which is the prospective lower jaw.

Craniofacial abnormalities are relatively common birth defects in humans. For example, cleft palate affects 1 in ~700 births, and it can lead to serious physical (eating difficulty, ear infection) and socio-psychological (speech, self-esteem) problems [3-5]. The process of palate development is very conserved between humans and mice, and thus studies from the latter have contributed greatly to our understanding of normal and abnormal palatogenesis [6,7]. In mice, the primary palate forms from the fusion of the frontonasal prominence and maxillary arch at the rostral end of the face around embryonic day (E) 10.5 (mouse gestation is 19 days). On the other hand, the secondary palate develops more caudally from the medial side of the maxillary arch. The secondary palate first appears as a bilateral outgrowth of the palatal shelves on either side of the tongue at ~ E11.5. Subsequently, the palatal shelves elongate vertically, elevate themselves into a horizontal position above the tongue, grow toward each other, and fuse at the midline at ~ E16.5 to complete the formation of the secondary palate. Perturbation in any of these steps can lead to cleft palate, and a large number of genes are involved in the tight regulation of each step [6-8].

LDB1 (LIM-domain binding protein 1, also known as NLI and CLIM2) encodes an evolutionarily conserved protein, found in organisms ranging from humans to nematodes [9]. LDB1 acts as an essential cofactor for various proteins, including LIM-domain homeodomain transcription factors and LIM-only (LMO) proteins [9-12]. In mice, *Ldb1* is ubiquitously expressed during development, and a global knockout of *Ldb1* caused mid-gestation lethality with severe defects, such as loss of the heart and anterior head [13]. In addition, tissue-specific deletions using a conditional knockout allele revealed that *Ldb1* is important in the development of the central nervous system (CNS), hematopoietic system, and limbs at later stages [14-16].

To elucidate the potential role of Ldb1 in craniofacial development, we generated a tissue-specific *Ldb1* mutant using Wnt1-Cre, which causes gene deletion in neural crest-derived cells [17,18]. We discovered that *Ldb1* plays an essential role in the morphogenesis of the secondary palate.

## Results

### Expression of *Ldb1* during craniofacial development

Although *Ldb1* is broadly expressed during development, it is not expressed at the same level everywhere. Therefore, we examined the expression of *Ldb1* during craniofacial development at E10.5 - E14.5, focusing on the developing palate. Prior to the current study, the information on the facial expression of *Ldb1* was available only at E10.5 [19]. For RNA in situ hybridization, we used an anti-sense probe against exons 5 through 9 of *Ldb1*, which are floxed in the conditional knockout allele [14]. We simultaneously performed in situ hybridization on the sections from control (*Ldb1*^*fl/-*^) and *Wnt1-Cre;Ldb1*^*fl/-*^ mutant embryos. This strategy allowed us to distinguish low levels of *Ldb1* transcript from non-specific background (because the former should disappear upon Cre-mediated deletion), and to verify efficient inactivation of *Ldb1* in the facial mesenchyme by Wnt1-Cre.

**Figure 1:**
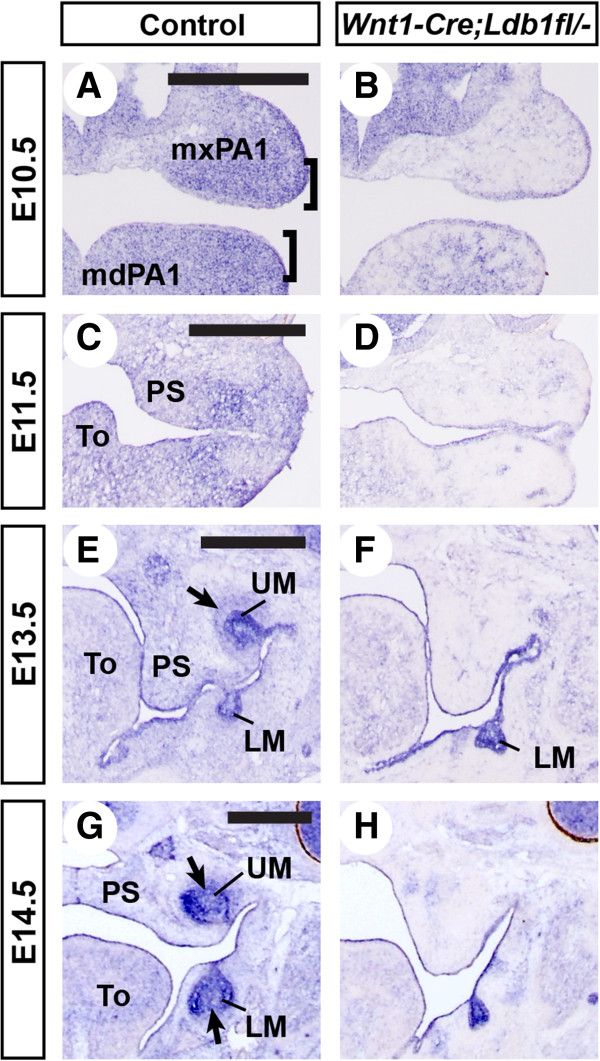
**Expression of*Ldb1*during palate development. (A-H)** Coronal sections of the heads were processed by RNA in situ hybridization for *Ldb1*, using a probe against exons 5–9. *Ldb1*^*fl/-*^ embryos were used as controls in this figure. Brackets in **A**, oral half of the first pharyngeal arch (PA1). Arrows in **E** and **G**, condensed dental mesenchyme. Abbreviations: LM, lower molar; mdPA1, mandibular arch; mxPA1, maxillary arch; PS, palatal shelf; To, tongue; UM, upper molar. Bar, 0.5 mm.

At E10.5, which is just before palate development begins, *Ldb1* was expressed broadly and strongly in the first pharyngeal arch mesenchyme in control embryos (Figure 1A). The mesenchymal expression of *Ldb1* was moderately higher in the oral half (brackets in Figure 1A) than in the aboral half, consistent with the positive regulation of *Ldb1* expression by FGF8 from the oral ectoderm [19]. Contrary to the earlier report [19], we also detected the expression of *Ldb1* in the epithelium of the first pharyngeal arch, although at a lower level than in the mesenchyme (Figure 1A).

Between E11.5 and E14.5, there was a reversal in the relative intensity of *Ldb1* expression in the mesenchyme compared with in the epithelium (Figure 1C-H). At E13.5 and E14.5, the expression in the mesenchyme was barely detectable except in the condensed dental mesenchyme (arrows in Figure 1E,G), whereas the expression in the epithelium, including the developing teeth, became more pronounced (Figure 1E-H). Throughout the stages examined, there was no obvious difference in the expression of *Ldb1* along the antero-posterior axis of the face (Additional file 1: Figure S1).

We confirmed that Wnt1-Cre caused deletion of *Ldb1* in most of the facial mesenchyme, except in a small, scattered group of cells (Figure 1B,D,F,H). This is consistent with the presence of mesoderm-derived cells in the mesenchyme [20].

### Neural crest-specific inactivation of *Ldb1* leads to cleft secondary palate

**Figure 2:**
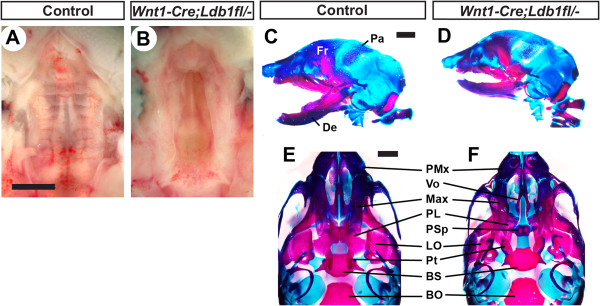
**Cleft secondary palate of*Wnt1-Cre;Ldb1***^***fl/-***^**mutants.** All the samples are E18.5. **A,B)** Whole mount view of the palate. The nose is toward the top. **C**-**F)** Skeleton staining for bone (red) and cartilage (blue). **C** and **D** are lateral views of the head. **E** and **F** show the palate area after the removal of the lower jaw, in the same orientation as in **A** and **B**. Note that the vomer (Vo) and presphenoid (PSp) in **F** are not visible in **E** because they are underneath the maxilla (Max) and palatine (PL) normally. Abbreviations: BO, basioccipital; BS, basisphenoid; De, dentary; Fr, frontal bone; LO, lamina obturans; Pa, parietal bone; PMx, premaxilla; Pt, pterygoid. Bar, 1 mm.

We found that all of the *Wnt1-Cre;Ldb1*^*fl/-*^ mutants had fully cleft secondary palate at birth (Figure 2A,B; N = 8). Skeletal preparations of the mutants showed no gross abnormality in the facial structures, apart from the defects in the maxilla and palatine bone associated with the cleft palate phenotype (Figure 2C-F). Consistent with the idea that the development of the face was not broadly affected in the *Wnt1-Cre;Ldb1*^*fl/-*^ mutants, they appeared indistinguishable from control embryos at E10.5, with neural crest-derived cells showing normal distribution in all of the facial primordia (Additional file 1: Figure S2).

### *Ldb1*-mutant palatal shelves have abnormal morphology and are impaired in reorientation

**Figure 3:**
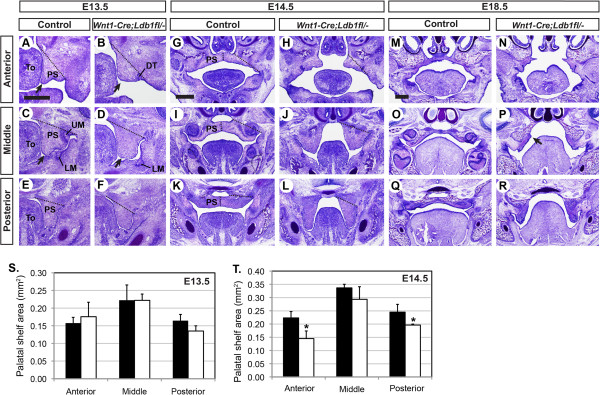
**Morphological and morphometric analyses of the palate in*Wnt1-Cre;Ldb1***^***fl/-***^**mutants. A**-**R)** Coronal sections of the heads were stained with cresyl violet. Arrows in **A**-**D**, the distal tip of the palatal shelf. Arrow in P, a protrusion on the medial side of the palatal shelf, considered an intermediate structure for palatal shelf elevation. Abbreviation: DT, diastema tooth. Bar, 0.5 mm. **S** and **T)** Quantitative analysis of the area of the palatal shelf. The boundary of the palatal shelf used for this measurement is indicated by the dotted lines in **A**-**L**. *: p < 0.05.

To determine which aspect of palatogenesis was disrupted in *Wnt1-Cre;Ldb1*^*fl/-*^ mutants, we examined the morphology of the mutant palate at various stages. We examined three positions along the antero-posterior axis of the palate, because there is heterogeniety in the genetic regulation of palate development along this axis. We found that from E13.5, the mutant palatal shelf had an abnormal shape in that it was more blunt and wider at the bottom compared with the controls (Figure 3A-F); this was more pronounced in the anterior and middle levels than in the posteior level (Figure 3A-D, arrows). The size of the mutant palatal shelves, measured as the area on the sections, was not significantly different from the controls at E13.5 (Figure 3A-F,S). However, from E14.5, the mutant palatal shelves were smaller than the controls in the anterior and posterior palate (Figure 3G-L,T). In addition, the re-orientation of the palatal shelf was impaired in *Wnt1-Cre;Ldb1*^*fl/-*^ mutants; at E14.5, the anterior palate was elevated above the tongue in both controls and *Ldb1* mutants (Figure 3G,H). However, the middle palate and the posterior palate remained vertical in the mutants at this stage (Figure 3J,L). Just before the birth (E18.5), the mutant middle palate appeared to have initiated the re-orientation as indicated by the medial protrusion (arrow in Figure 3P), which is considered an intermediate structure for palatal shelf elevation [21,22]. On the other hand, the posterior palate failed to elevate even at this late stage (Figure 3R).

Tissue sections also revealed that *Wnt1-Cre;Ldb1*^*fl/-*^ mutants had abnormal dentition, including loss of molars and appearance of diastema teeth on the upper jaw, and developmental arrest of lower molars. The details of the tooth phenotype will be described elsewhere.

### Defects intrinsic to the palate are responsible for failed elevation of *Ldb1*-mutant posterior palate

Multiple mouse mutants had been reported to have defects in palatal shelf elevation (see Discussion for details). One of the common causes of a failure in palatal shelf elevation is the interference by the tongue, when the tongue is not sufficiently depressed within the oral cavity. We noticed that the tongue of *Wnt1-Cre;Ldb1*^*fl/-*^ mutants was abnormally tall (Figure 3L,R). Therefore, we asked whether the defect in palatal shelf elevation was secondary to the tongue phenotype.

**Figure 4:**
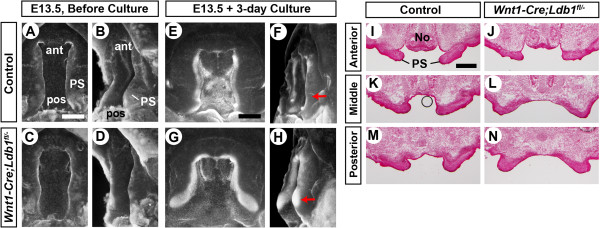
**Removing the lower jaw does not restore the reorientation of the palatal shelves in*Wnt1-Cre;Ldb1***^***fl/-***^**mutants.** The upper jaw region from E13.5 embryos, before culture **(A-D)** and after 3-day culture in the absence of the lower jaw and the tongue **(E-N)**. **A**-**H** are fluorescent pictures of the heads processed by whole mount DAPI staining. **(A,C,E,G)** Intra-oral views directly into the palate. **(B,D,F,H)** Oblique views showing the profile of the palatal shelf. Red arrows in **F** and **H** point to the posterior palatae. **(I-N)** Coronal sections were stained with Nuclear Fast Red. Abbreviations: ant, anterior; pos, posterior; No, nose. Bar, 0.5 mm.

To answer this question, we dissected embryos at E13.5, before the palatal shelves were elevated. We removed the tongue and the lower jaw from their heads, and cultured the heads rotating in a culture tube for 3 days. In all of the control embryos, one or both of the palatal shelves became horizontal throughout the antero-posterior axis after the culture (Figure 4A,B,E,F,I,K,M; N = 10). In contrast, in all of the *Ldb1*-mutant embryos, both palatal shelves remained vertical in the middle and posterior domains (Figure 4C,D,G,H,L,N; N = 8). The mutant anterior palate was horizontal after the culture (Figure 4J), which is consistent with the in vivo finding (Figure 3). Hence, we concluded that the defect in middle/posterior palatal shelf elevation in *Wnt1-Cre;Ldb1*^*fl/-*^ mutants is not due to the mechanical hindrance by the tongue.

### Localized abnormality in cell proliferation in *Wnt1-Cre;Ldb1*^*fl/-*^ mutant palatal shelves

Since the palatal shelves of *Wnt1-Cre;Ldb1*^*fl/-*^ mutants were moderately smaller than the controls at E14.5 (Figure 3T), we asked whether a change in cell proliferation or cell death could explain this phenotype.

**Figure 5:**
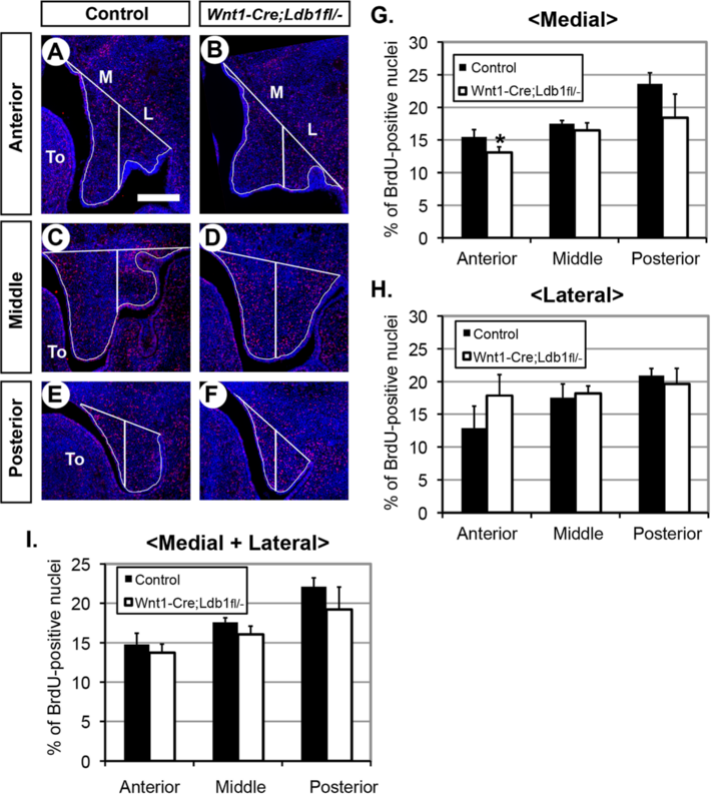
**Cell proliferation analysis. (A-F)** Coronal sections of the heads from E13.5 embryos were processed with immunofluorescence for BrdU (red) and counter-stained with DAPI for nuclei (blue). **(G-H)** Quantitative analyses of the mitotic index in the palatal shelf, in the medial (M), lateral (L) or combined **(I)** areas as demarcated by white lines in **A**-**F**. *, p > 0.05. Bar, 0.2 mm.

We labeled proliferating cells with bromodeoxyuridine (BrdU) at E13.5, performed immunofluorescence with anti-BrdU antibody on sections, and calculated the percentage of mitotic cells in the palate mesenchyme (Figure 5). The sections of the palatal shelves were divided into medial and lateral areas (Figure 5A-F), and the mitotic index was calculated for each area and for the total area (Figure 5G,H,I). From the comparison between the controls and *Wnt1-Cre;Ldb1*^*fl/-*^ mutants, the only statistically significant difference (p < 0.05) was found in the medial area of the anterior palate; this region showed reduced cell proliferation in the mutants (Figure 5G). Although the lateral area of the anterior palate had higher average mitotic index in the mutants than in controls, this difference was not statistically significant (Figure 5H). When the total area of the palatal shelf was considered, there was no significant difference in cell proliferation at any position along the anterio-posterior axis (Figure 5I). Together, we conclude that the small decrease in the cell proliferation in the anterior-medial palate contributed to the hypoplasia of this region in *Wnt1-Cre;Ldb1*^*fl/-*^ mutants (Figure 3).

We also stained the palate sections with an antibody against cleaved caspase-3, to label apoptotic cells. There were very few dying cells in the palate mesenchyme of both controls and *Ldb1* mutants, and no difference was apparent between the genotypes (Additional file 1: Figure S3).

### Expression of *Wnt5a*, *Osr2* and *Pax9*, which regulate palatal shelf elevation, is altered in the *Ldb1*-mutant palate

Since LDB1 serves as a cofactor for multiple transcription factors, it is likely that LDB1 regulates the expression of other genes in the developing palate. Therefore, we asked whether the inactivation of *Ldb1* leads to a change in the expression of genes important for palate development. In particular, we focused on the genes that are involved in reorientation of the palatal shelf, the process most affected in *Wnt1-Cre;Ldb1*^*fl/-*^ mutants. *Wnt5a, Osr2*, *Pax9* and *Zfhx1a* have been implicated in palatal shelf elevation because the mutation of each gene in mice led to delay or failure in elevation [23-27]. *Pdgfra*, expressed in the palatal mesenchyme, is also important in palatal shelf elevation as indicated by the delayed elevation in mouse mutants of *Pdgfc*, encoding a ligand for *Pdgfra*[28]*.*

**Figure 6:**
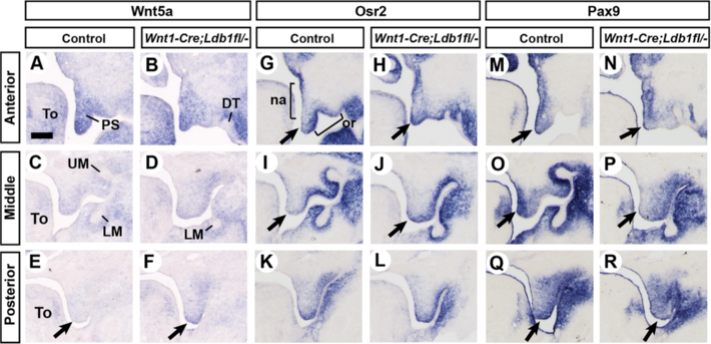
**Altered expression of*Wnt5a, Osr2*and*Pax9*in*Wnt1-Cre;Ldb1***^***fl/-***^**mutant palatal shelf. (A-R)** Coronal sections of the heads from E13.5 embryos were processed by RNA in situ hybridization. Arrows in **E** and **F**, ectopic expression of *Wnt5a* in the posterior palate. Arrows in **G**-**J**, up-regulation of *Osr2* in the distal tip of the *Ldb1* mutant palatal shelf. Arrows in **M**-**R**, down-regulation of *Pax9* in the *Ldb1* mutant palatal shelf. Abbreviations: na, nasal domain of the palatal shelf; or, oral domain of the palatal shelf. Bar, 0.2 mm.

We found changes in the expression of *Wnt5a*, *Osr2* and *Pax9* in *Wnt1-Cre;Ldb1*^*fl/-*^ mutant palate. WNT5A was shown to regulate cell migration in the palatal shelf; specifically, it attracted mesenchyeme cells [23]. During normal palatogenesis, *Wnt5a* is expressed strongly in the anterior palate but minimally in the posterior palate at E13.5 (Figure 6A,C,E). However, in the *Ldb1* mutants, there was ectopic expression of *Wnt5a* at the distal tip of the posterior palatal shelf (arrows in Figure 6E,F). Chemoattraction of the palatal mesenchyme cells toward this location could disrupt elevation of the palatal shelves (see Discussion). *Osr2* is normally expressed with asymmetric intensity in the anterior and middle palate, weaker on the nasal side and the distal tip of the palatal shelf (arrows in Figure 6G,I), and stronger on the oral side (Figure 6G,I [24]). In the *Ldb1* mutants, *Osr2* expression was up-regulated in the distal tip of the anterior and middle palate (arrows in Figure 6G-J). On the other hand, *Pax9* was down-regulated in the *Ldb1* mutants in the distal tip (arrows in Figure 6M-R) and the nasal side of the middle palate (Figure 6O,P). The expression patterns of *Zfhx1a* and *Pdgfra* were normal in *Wnt1-Cre;Ldb1*^*fl/-*^ mutant palate (Additional file 1: Figure S4).

## Discussion

In this paper, we investigated the role of *Ldb1* in mammalian craniofacial development. LDB1 can bind to various proteins, including 12 LIM-domain homeodomain transcription factors and 4 LIM only (LMO) proteins in mammals, and is thought to act as a cofactor modulating the activities of these proteins [9]. Although Ldb1 was shown to play crucial roles in neuronal differentiation in the brain and spinal cord, erythropoiesis, and limb development [9,14-16], its importance in craniofacial development had been unknown. We found that the function of LDB1 is essential for normal development of the secondary palate, in particular, for the reorientation of the palatal shelves.

The details of the molecular and cellular processes of palatal shelf elevation remain to be elucidated, but it is thought to involve two mechanisms: a rapid “swinging” of the palatal shelf from vertical to horizontal position is a favored model for the anterior palate, whereas tissue remodeling involving the “flow” of the cells from the ventral to the medial side of the palatal shelf is supported by histological studies in the posterior palate [21,22]. In case of *Wnt1-Cre;Ldb1*^*fl/-*^ mutants, the anterior palate elevated normally while the posterior palate failed to elevate, even though the anterior palate suffered from more severe growth deficiency than the posterior palate. Therefore, our result supports the notion that two distinct mechanisms regulate the reorientation of the palatal shelf along the antero-posterior axis, and indicates that only the mechanism for the posterior palate was affected in *Wnt1-Cre;Ldb1*^*fl/-*^ mutants.

Palatal defects have been described in mouse mutants of more than 100 genes, and the delay or failure in palatal shelf elevation was noted in many of them [6,7,29]. Inactivation of *Pdgfc*, *Osr2, Pax9, Zfhx1a,* and *Adamts20* caused a 1 ~ 2 day delay in palatal shelf elevation, so did gain-of-function of *Fgfr2* and *Bmpr1a*[24-28,30-32]. On the other hand, inactivation of *Jag2, Fgf10, Spry2, Wnt5a, Gsk3b, Tbx1, Tak1* and *Fgfr1* led to failure in palatal shelf elevation, at least within the developmental stages examined in each study [23,33-41]. We found that the posterior palate of *Wnt1-Cre;Ldb1*^*fl/-*^ mutants remained vertical up to E18.5, the last stage when we can collect the mutants because they die shortly after birth, and thus the phenotype was a failure rather than a delay of posterior palatal shelf elevation.

Among the examples with failed elevation of palatal shelves, the mutants for *Jag2, Fgf10,* and *Tbx1* showed abnormal fusion between the palatal epithelium and oral or tongue epithelium, which likely contributed the defect [33,34,38]. In *Tak1* mutants, the palate defect was due to the mechanical hindrance by the malformed tongue [39,41]. In contrast, in the mutants of *Wnt5a, Spry2, Gsk3b,* and *Fgfr1*, the palatal elevation defect was attributed to changes intrinsic to the palatal shelves, including abnormal levels of cell proliferation and cell death [23,36,37,40]. Furthermore, *Wnt5a* mutation affected migration of the mesenchyme cells in the palatal shelf; He et al. [23] demonstrated that there was directional migration of the cells in the developing palate, and that WNT5A acted as a chemoattractant for palatal mesenchyme cells.

In the current study, we ruled out the interference by the tongue as a cause of the palate defect in *Wnt1-Cre;Ldb1*^*fl/-*^ mutants. We did not find evidence of aberrant adhesion between the *Ldb1* mutant palatal shelf and the tongue or oral epithelium. Although there was a localized decrease in the cell proliferation in *Wnt1-Cre;Ldb1*^*fl/-*^ mutant palate, it was only found in the anterior palate, which did not exhibit reorientation defect. On the other hand, we did find ectopic expression of *Wnt5a* at the distal tip of the posterior palatal shelf in *Wnt1-Cre;Ldb1*^*fl/-*^ mutants. This ectopic WNT5A could pull the palatal mesenchyme cells toward the distal end of the palatal shelf, preventing the cells from “flowing” to the medial side, which is thought to be integral to the reorientation of the palatal shelves in the posterior domain. Further experiments are necessary to test this hypothesis.

The up-regulation of *Osr2* and down-regulation of *Pax9* could also contribute to the palatal defect of the *Ldb1* mutants. However, the changes in *Osr2* and *Pax9* expression were more pronounced in the middle palate than in the posterior palate, and thus did not correlate with the relative severities of the elevation defect. Furthermore, while the morphologies of the anterior and middle palate are very similar between *Pax9*^*−/−*^ mutants and *Wnt1-Cre;Ldb1*^*fl/-*^ mutants (such as the absence of the indentation medial to the upper molar) [26,27], the phenotypes differ in the posterior palate. In *Pax9*^*−/−*^ mutants, the posterior palate became horizontal by E15.5, indicating that there is a delay but not a failure in palatal elevation [27]. Also, *Pax9*^*−/−*^ mutants showed a significant decrease in cell proliferation in the posterior palate at E13.5, and consequently, suffered from much more severe hypoplasia of this region than *Wnt1-Cre;Ldb1*^*fl/-*^ mutants at E14.5 [27]. Therefore, it is possible that the delay in palatal elevation in *Pax9*^*−/−*^ mutants is secondary to growth deficiency, whereas in *Wnt1-Cre;Ldb1*^*fl/-*^ mutants, the process of palatal elevation is directly affected.

Among the LIM domain proteins, LHX6 and LHX8 play important roles in the development of the secondary palate, and thus they are the most likely partners of LDB1 for its role in palatogenesis. Mutation of *Lhx8* in mice resulted in cleft secondary palate with partial penetrance, in which the horizontal growth and/or fusion of the palatal shelves were affected [42]. Although inactivation of *Lhx6* did not cause overt palatal defects [43], simultaneous inactivation of *Lhx6* and *Lhx8* resulted in fully penetrant cleft palate, indicating that the two genes have overlapping functions [44].

## Conclusions

We established that *Ldb1* in the facial mesenchyme is essential for normal development of the secondary palate. Inactivation of *Ldb1* resulted in changes intrinsic to the palatal shelves that led to the failure in reorientation of the posterior palate. In addition, our results suggest that LDB1 is involved in regulating the expression of *Wnt5a, Osr2* and *Pax9* in the palate, genes that have been implicated in palatal shelf elevation.

## Methods

### Animals

All the experiments involving animals were performed with the approval from New York University Institutional Animal Care and Use Committee. *Wnt1-Cre*, *Ldb1*^*+/−*^ and *Ldb1*^*fl/fl*^ mice have been described [13,14,17]. The tissue-specific *Ldb1* mutant embryos (*Wnt1-Cre;Ldb1*^*fl/-*^) were obtained from the crosses between *Ldb1*^*fl/fl*^ and *Wnt1-Cre;Ldb1*^*+/−*^. The embryos were genotyped by PCR using DNA from the tail. Littermates of all the other genotypes from the above cross were indistinguishable from wild type embryos, and thus they were used as controls without distinction unless otherwise specified.

### Skeleton staining, Cresyl Violet staining, RNA in situ hybridization

For skeleton staining, the skin was removed from E18.5 embryos, and the skeleton was stained with Alcian blue and Alizarin red as described previously [43]. To prepare frozen sections, the embryos were fixed in 4% paraformaldehyde in PBS overnight, washed with PBS, and cryoprotected in 10% sucrose for 1 day then in 20% sucrose for 1 day, and embedded in OCT (Tissue-tek). The sections were prepared at 12 μm ~ 20 μm depending on the age of the embryo. All the sections used in this study are in the coronal plane. The frozen sections were stained with 0.1% cresyl violet solution as described [43] to visualize tissue morphology. For RNA in situ hybridization, the frozen sections were hybridized with Digoxigenin-labeled RNA probes as described [43].

### Morphometric analysis of the size of the palatal shelf

The area of the palatal shelf was measured from the photographs of cresyl violet-stained sections using ImageJ program as described [43]. Two palatal shelves were measured from each embryo, and measurements from three mutants and three controls were used for statistical analysis (Student’s t-test).

### Detection of cell proliferation and apoptosis

To detect proliferating cells, a pregnant female was injected intraperitoneally with BrdU solution (Invitrogen) 2 hours before harvesting embryos at E13.5. Immunofluorescence with anti-BrdU antibody (Abcam, rat monoclonal, 1:200) was performed following the manufacturer’s protocol, and DAPI was used to stain nuclei. To calculate the percentage of dividing cells, we first counted the total number of nuclei in a defined area from DAPI images, by automated counting using ImageJ plug-in followed by manual confirmation. Then the number of BrdU-positive cells from the same area was counted manually, and this number was divided by the total number of nuclei. The palatal shelf was divided into medial and lateral domains on the coronal sections, by a vertical line drawn from the mid-point of the border that separates the palatal shelf and the rest of the upper jaw (see Figure 5A-F). Two palatal shelves from each embryo, and three mutant and three control embryos were analyzed. Student’s t-test was used to determine whether the difference was statistically significant (= p < 0.05). To detect apoptotic cells, immunofluorescence with anti-caspase3 antibody (Cell Signaling Technology) was performed as previously described [43].

### Culture of the palate in rotating tubes

The embryos were dissected at E13.5 and decapitated in PBS, and the lower jaw and the top of the head (calvaria region) were further removed. The remaining upper jaw region was placed in a glass culture tube with 1.5 ml of CO_2_-independent medium (Life Technologies) supplemented with 20% fetal bovine serum and antibiotics-antimycotics (Life Technologies), and cultured on a rotisserie inside a 37°C incubator. After 3 days, the upper jaw was rinsed with PBS and fixed with 4% paraformaldehyde, and processed for frozen sectioning as described above, or for whole-mount DAPI staining following a published protocol [45].

## Competing interests

The authors declare that they have no competing interests.

## Authors’ contributions

JJ designed the project, analyzed data, and drafted the manuscript. YZ provided the initial idea for the project and the reagent. AA, JC and NS performed the cell proliferation and apoptosis assays and analyzed the data. ALM, VC and JC performed the rotating tube palate culture experiment and analyzed the data. AA collected and genotyped embryos, prepared tissue sections, and performed cresyl violet staining and morphometric analysis. JC performed skeletal staining, and JJ and JC performed RNA in situ hybridization. All authors read and approved final manuscript.

## Supplementary Material

Additional file 1: Figure S1 Expression of *Ldb1* during palate development. Coronal sections of the heads were processed by RNA in situ hybridization for *Ldb1*, using a probe against exons 5–9. *Ldb1fl/-*embryos were used as controls in this figure. Abbreviations: DT, diastema tooth; LM, lower molar; mdPA1, mandibular arch; mxPA1, maxillary arch; PS, palatal shelf; To, tongue; UM, upper molar. Bar, 0.5 mm. **Figure S2.** Distribution of neural crest-derived cells in the developing face. The neural crestderived cells (green) were visualized using *Wnt1-Cre* and *R26REYFP* reporter system [18]. The exact genotypes of the embryos are *Wnt1-Cre;Ldb1fl/+;R26REYFP/+* for the control and *Wnt1-Cre;Ldb1fl/-;R26REYFP/+* for the mutant. The distribution of neural crest-derived cells appeared normal in the mutant. Bar, 0.5 mm. **Figure S3.** Detection of apoptotic cells in the palatal shelf. Coronal sections of the heads from E13.5 embryos were processed with immunofluorescence for cleaved caspase 3 (red) and counter-stained with DAPI for nuclei (blue). The white lines demarcate the palatal shelves. There were very few apoptotic cells in the palate in the embryos of either genotype. Bar, 0.5 mm. **Figure S4.** Expression of *Zfhx1a* and *Pdgfra*. Coronal sections of the heads from E13.5 embryos were processed by RNA in situ hybridization. The expression of *Zfhx1a* and *Pdgfra* was not altered in *Wnt1-Cre;Ldb1fl/-* mutant palatal shelf. Bar, 0.2 mm. Click here for file
