# Supplementary figures and images for: Neural crest-specific deletion of Ldb1 leads to cleft secondary palate with impaired palatal shelf elevation
Source: BMC Dev Biol. 2014 Jan 17;14:3. doi: 10.1186/1471-213X-14-3 (PMC3899388; doi:10.1186/1471-213X-14-3)

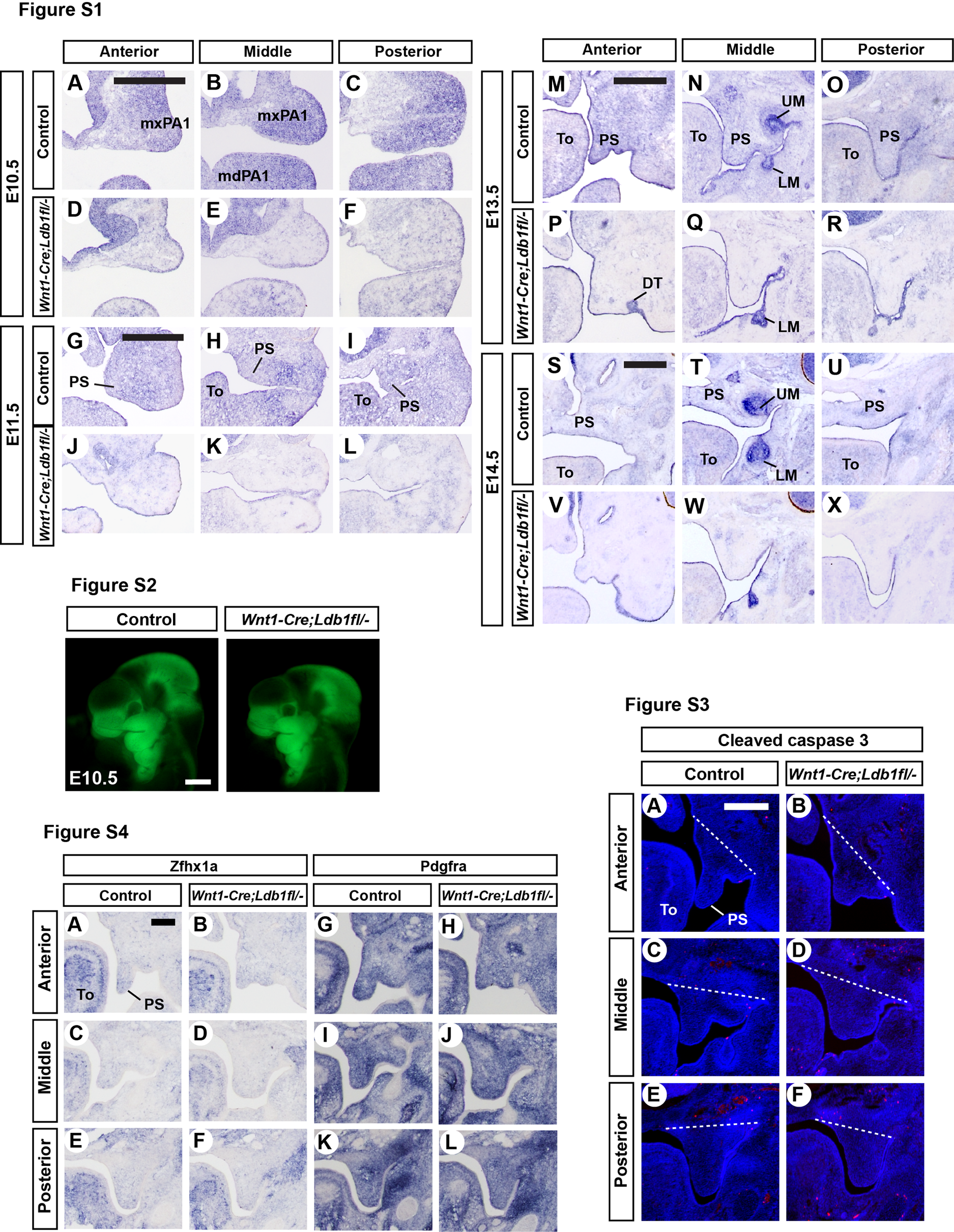

Supplement: Additional file 1: Figure S1 — Expression of Ldb1 during palate development. Coronal sections of the heads were processed by RNA in situ hybridization for Ldb1, using a probe against exons 5–9. Ldb1fl/-embryos were used as controls in this figure. Abbreviations: DT, diastema tooth; LM, lower molar; mdPA1, mandibular arch; mxPA1, maxillary arch; PS, palatal shelf; To, tongue; UM, upper molar. Bar, 0.5 mm. Figure S2. Distribution of neural crest-derived cells in the developing face. The neural crestderived cells (green) were visualized using Wnt1-Cre and R26REYFP reporter system [18]. The exact genotypes of the embryos are Wnt1-Cre;Ldb1fl/+;R26REYFP/+ for the control and Wnt1-Cre;Ldb1fl/-;R26REYFP/+ for the mutant. The distribution of neural crest-derived cells appeared normal in the mutant. Bar, 0.5 mm. Figure S3. Detection of apoptotic cells in the palatal shelf. Coronal sections of the heads from E13.5 embryos were processed with immunofluorescence for cleaved caspase 3 (red) and counter-stained with DAPI for nuclei (blue). The white lines demarcate the palatal shelves. There were very few apoptotic cells in the palate in the embryos of either genotype. Bar, 0.5 mm. Figure S4. Expression of Zfhx1a and Pdgfra. Coronal sections of the heads from E13.5 embryos were processed by RNA in situ hybridization. The expression of Zfhx1a and Pdgfra was not altered in Wnt1-Cre;Ldb1fl/- mutant palatal shelf. Bar, 0.2 mm. [file 1471-213X-14-3-S1.tiff]
